# Biphasic Electrical Stimulation of Schwann Cells on Conducting Polymer-Coated Carbon Microfibers

**DOI:** 10.3390/ijms26168102

**Published:** 2025-08-21

**Authors:** Alexandra Alves-Sampaio, Jorge E. Collazos-Castro

**Affiliations:** Neural Repair and Biomaterials Laboratory, Hospital Nacional de Parapléjicos (SESCAM), Finca La Peraleda S-N, 45071 Toledo, Spain; aalves@sescam.jccm.es

**Keywords:** microfiber, conducting polymer, PEDOT, carbon microfiber, Schwann cell, electrical stimulation, peripheral nerve, growth factor, regeneration

## Abstract

Electroactive biomaterials are a key emerging technology for the treatment of neural damage. Conducting polymer-coated carbon microfibers are particularly useful for this application because they provide directional support for cell growth and tissue repair and simultaneously allow for ultrasensitive recording and stimulation of neural activity. Here, we report in vitro experiments investigating the biology of Schwann cells (SCs), a major player in peripheral nerve regeneration, on electroconducting microfibers. The optimal molecular composition of the cell substrate and cell culture medium was studied for SCs dissociated from rat and pig peripheral nerves. The substrate molecules were then attached to carbon microfibers coated with poly (3,4-ethylenedioxythiophene) doped with poly [(4-styrenesulfonic acid)-*co*-(maleic acid)] (PCMFs), which served as an electroactive scaffold for culturing nerve explants. Biphasic electrical stimulation (ES) was applied through the microfibers, and its effects on cell proliferation and migration were assessed in different cell culture media. Rodent and porcine SCs avidly migrated on PCMFs functionalized with a complex of poly-L-lysine, heparin, basic fibroblast growth factor, and fibronectin. Serum and forskolin/heregulin increased, by two-fold and four-fold, the number of SCs on PCMFs, respectively, and ES further doubled cell numbers without favoring fibroblast proliferation. ES additionally increased SC migration. These results provide a baseline for using biofunctionalized PCMFs in peripheral nerve repair.

## 1. Introduction

Peripheral nerve injury (PNI) is a relatively common and debilitating condition leading to loss of motor function and sensory capacity. Overall, about 130 new PNI cases occur per million persons a year [1]. The site of injury is most frequently located in the upper extremity, and poor function recovery accompanied by neuropathic pain occurs in more than 50% of cases [2]. Furthermore, the majority of injuries occur in young people between 25 and 49 years [3,4]. As a consequence, a life-long negative impact on the psychosocial well-being and employment is observed [2,5].

Compared to the central nervous system (CNS), peripheral nerves have a remarkable potential for spontaneous regeneration in adult mammals [6]. In addition to the intrinsic ability of some types of adult peripheral neurons for axonal regrowth [7], Schwann cells (SCs) significantly contribute to nerve repair [8,9]. SCs proliferate and migrate in response to nerve injury, providing trophic support and guidance to regenerating axons [8,9,10]. Despite this potential, traumatic nerve injuries frequently produce large (>1 cm) neural tissue gaps that preclude successful axonal regeneration and functional recovery [6,11,12]. Nerve autografts, allografts, and devices used in the clinical setting to bridge the gap have achieved some therapeutic success but still show several limitations [6,11,12]. Devices mostly consist of hollow, single-channel conduits made of extracellular matrix proteins or synthetic polymers [11,12,13]. Although they improve nerve reconstruction, important functional deficits persist in the treated patients due to inaccurate and slow muscle fiber reinnervation and incomplete regeneration of some neuronal types [6,7,10,11,12,13,14]. Consequently, there is a clinical need for advanced biomaterials and devices able to provide mechanical support, trophic stimuli, and directional guidance to the injured nerve. In this regard, more advanced conduits filled with aligned extracellular matrix proteins, or with nanofibers/microfibers made of natural or synthetic polymers, implanted alone or further loaded with growth factors or cells, have shown superior results to bridge peripheral nerve gaps in experimental animals [11,15,16,17,18,19].

Peripheral nerve regeneration may be enhanced in rodents and humans by using brief (1 h), low-frequency (20 Hz) electrical stimulation (ES) applied proximally to the nerve injury site [7]. In rat PNI models, this regime of ES increases neuronal expression and responsivity to neurotrophins [20] and accelerates neuronal upregulation of growth-associated protein 43 (GAP-43) [21]. Likewise, voltage pulses at 1–3 Hz for 30 min enhance the production of neurotrophic factors by rat SCs in vitro [22,23], whereas seven days of current-controlled biphasic ES (100 Hz, 10 µA/cm^2^), applied through gold cell culture substrates, doubles SC proliferation and growth factor release in vitro [24]. Accordingly, there is increasing interest in combining ES and nerve conduits to improve peripheral nerve regeneration, and compelling evidence exists for the feasibility and effectiveness of this approach in rodents [25,26,27]. In the most common experimental paradigm, channels made of non-conducting materials are implanted to bridge nerve lesions and electricity is administered through electrodes placed at the nerve stumps or over the skin. Nevertheless, electroconducting materials, such as polypyrrole, polyaniline, or carbon nanotubes, have also been used [25,26,27,28,29,30]. For instance, PNI bridging with polypyrrole/silk fibroin conduits, together with ES (3 V at 20 Hz in eight sessions of 1 h/day) applied via metallic wires secured to the conduit ends, augmented about 10% the number of axons regenerating through a peripheral nerve gap in rats [25]. However, in this study, the electrical conductivity of the polypyrrole composite was very low (1.5 S/m), likely precluding electric current flow through the lesion and, thus, limiting its effectiveness.

From the group of conducting polymers, poly (3,4-ethylenedioxythiophene) (PEDOT) and its copolymers have superior electrochemical properties [31,32]. Additionally, several methods have been developed for PEDOT biofunctionalization, i.e., for modification of the materials with molecules to enhance or control their interactions with biological cells [32,33,34,35,36,37]. In this regard, we propose the use of biofunctionalized PEDOT-coated carbon microfibers (PCMFs) as implantable scaffolding material for neural repair. Seven-micron carbon microfibers of high electrical conductivity (5.4 × 10^4^ S/m) were coated with PEDOT doped with poly [(4-styrenesulfonic acid)-*co*-(maleic acid)] (PSS-*co*-MA) and subsequently with N-Cadherin or L1 to facilitate direct cell attachment and axonal elongation from cerebral cortex neurons [33], or with multilayers of poly-L-lysine (PLL), heparin, basic fibroblast growth factor (bFGF), and fibronectin (FN), which promoted oligodendrocyte and astrocyte precursor cell migration and, thus, indirectly facilitated axonal elongation [35]. The latter biofunctionalized microfibers were implanted in the damaged spinal cord in rats [38], mice [39,40], and pigs [41], providing conclusive evidence that they can potentiate and guide neural regrowth across the lesion. These results, together with the positive effects of ES on peripheral nerve regrowth [7,25,26,27,28,29,30], suggest that biofunctionalized PCMFs can also meet the need of highly electroconducting biomaterials for the treatment of PNI. In this case, the microfibers will support long-distance migration of SCs accompanied by axonal regrowth, while efficient ES will accelerate and enhance the reparative responses and the specificity of target reinnervation, thus improving the functional outcomes [7,42,43]. As a first step to achieve this goal, the present work investigated the suitability of PCMFs for supporting SCs migrating from rodent and porcine peripheral nerves in vitro. Additionally, we studied the synergisms that biphasic ES, applied through the PCMFs, may have with biomolecules at the cell substrate or in the cell culture medium for promoting cell proliferation and migration.

## 2. Results

### 2.1. Optimal SC Substrate Coating

The appropriateness of different cell substrate coatings for expanding cells dissociated from rat peripheral nerves was first investigated by counting the total cells grown for 3 days on standard plastic surfaces. Cells were seeded at relatively low density (5000 cells/cm^2^) to prevent a fast confluence and, thus, facilitate the quantification of biological responses in each experimental paradigm. Before dissociating the tissue for cell plating, the nerve fascicles were cut in 2 mm long pieces and incubated for six days in cell culture medium (Section 4.4.1). This initial incubation of the nerves, hereafter referred to as the “preconditioning” step, allows Wallerian degeneration to proceed and increases the ratio of SCs to fibroblasts by favoring fibroblast migration out of the nerves and by stimulating SC proliferation [44,45]. For these experiments, the cell culture medium comprised Neurobasal with L-glutamine, Penicillin-Streptomycin, Gentamicin, and B27 (abbreviated as NB), fetal bovine serum (FBS), forskolin and human heregulin β-1 (FH), at the concentrations provided in Section 4.3. This culture medium composition will be called NB/FBS/FH throughout this manuscript. Considering the usefulness of pig SCs for translational research on human neurotherapeutics [44], the same experiments were performed using porcine nerves. As described below, the cells from both species behaved similarly when cultured on the same substrates.

**Figure 1:**
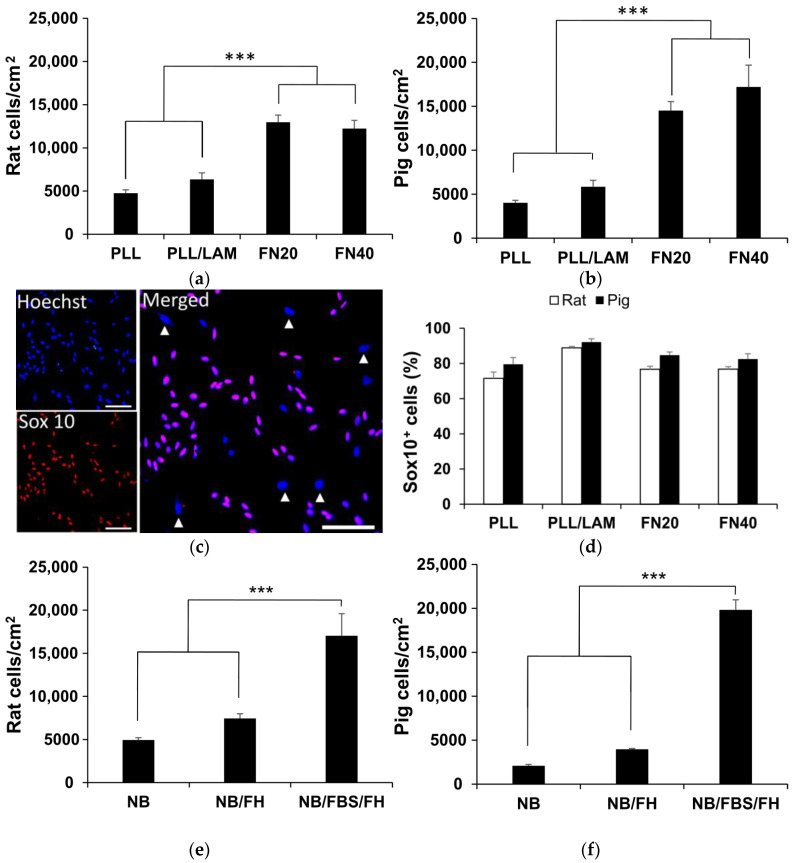
Growth of peripheral nerve cells on plastic cell culture plates with different surface coatings and cell culture media. Cells were seeded at 5000/cm^2^ and fixed after three days in vitro. (**a**,**b**) Total cell counts for rat (**a**) and pig (**b**) cells achieved on the indicated substrates, using the same NB/FBS/FH cell culture medium. FN20 and FN40 are used as abbreviations for the substrate coating comprising PLL/heparin/bFGF/fibronectin, with fibronectin used at 20 µg/mL or 40 µg/mL, respectively. (**c**) Representative image of cell nuclei quantified in (**a**), with SCs nuclei identified by Sox10 expression (double stained in red and blue colors). White arrowheads signal large cell nuclei from presumptive fibroblasts negative for Sox10. (**d**) Percentage of Sox10-positive rat and pig cells across the different substrates. (**e**,**f**) Total cell counts for rat (**e**) and pig (**f**) cells achieved in different cell culture media. Cells in (**e**,**f**) were grown on PLL/heparin/bFGF/fibronectin (40 µg/mL)-coated surfaces. Data represent the mean ± SEM. Statistical analysis was performed using two-way ANOVA and Holm–Sidak post hoc test. *** *p* < 0.001. Scale bar in (**c**): 100 µm.

Molecules coating the plastic substrate potently influenced the proliferation of adhered peripheral nerve cells. When only PLL was present, cells effectively attached but barely divided, and, therefore, at 3 days in vitro (DIV), the cultures had almost the same number of cells originally plated (i.e., 5000 cells/cm^2^) (Figure 1a,b). Addition of laminin on PLL produced no significant enhancement in cell numbers; in contrast, rodent cells multiplied between two- and three-times on PLL/heparin/bFGF/fibronectin-coated plates (Figure 1a), and porcine cells increased up to four-fold in the same condition (Figure 1b).

**Figure 2:**
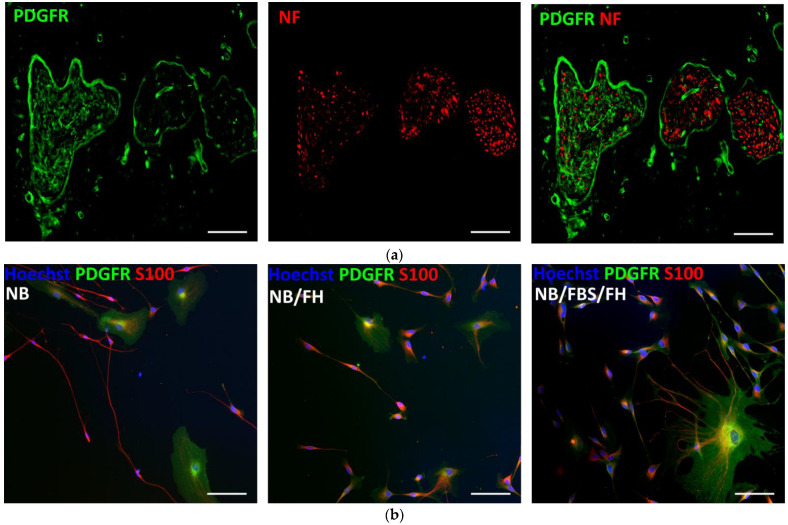
Immunofluorescent staining for platelet-derived growth factor receptor (PDGFR) in undamaged peripheral nerves (**a**), and in cell cultures from dissociated, preconditioned nerves (**b**). (**a**) Transverse 10 μm nerve sections stained for PDGFR (green) and neurofilament (NF, red). The endoneurium and the perineurium surround axonal fascicles and show intense PDGFR immunoreactivity. (**b**) Fluorescence microscopy images from representative cultures of dissociated nerve cells cultured on PLL/heparin/bFGF/fibronectin and different cell culture media. PDGFR (green) is predominantly expressed in fibroblast-like cells, which are larger and morphologically distinct from the smaller, bipolar Schwann cells. The latter shows strong immunoreactivity for S100 (red). Scale bars, 100 µm.

The transcription factor Sox10 is required for Schwann cell specification during development and also for maintaining Schwann cell identity in later stages [46]. The fact that Sox10 is not expressed in fibroblasts makes it a highly reliable marker for identification of SCs in peripheral nerves and cell cultures [47]. We used Sox10 immunocytochemistry for assessing cell purity on the different substrates. SCs displayed relatively small ellipsoidal nuclei positive for Sox10, clearly distinguishable from the Sox10-negative nuclei (Figure 1c). The latter were frequently of large size, as expected for fibroblasts (Figure 1c and Figure 2). Assessed as the percentage of Sox10^+^ cells, the purity of SCs was about 90% on PLL/Laminin-coated plates, whether harvested from rodent or porcine nerves (Figure 1d). SC purity tended to be somewhat lower (80%) on PLL/heparin/bFGF/fibronectin, although the difference between both substrates was not statistically significant. Because of its much higher efficiency for expanding rodent and porcine SCs, we selected PLL/heparin/bFGF/fibronectin for subsequent studies.

### 2.2. Essential Components of the SC Culture Medium

After comparing the substrate coatings, we assessed if FH and/or FBS in the cell culture medium are still necessary to achieve efficient cell growth when the plates are coated with the multimolecular complex of PLL/heparin/bFGF/fibronectin. This turned out to be the case. When the cell culture medium comprised only NB, most plated rat cells survived by 3 DIV without increasing in cell number, whereas less than half of the plated porcine cells survived by 3 DIV, and those alive showed small cytoplasm with short cell processes. NB supplementation with forskolin and heregulin, two well-known pro-survival and mitogenic molecules for SCs [44,45,48,49], favored the proliferation of rodent cells and the survival of porcine cells, increasing cell counts by 50% and 91%, respectively (Figure 1e,f). In addition, the cells showed healthy appearance at 3 DIV. Nevertheless, further addition of 10% FBS to the NB/FH medium allowed rodent cells to multiply by three- to four-fold in the same period (Figure 1e), and this effect was even more remarkable in porcine cells (Figure 1f). Thus, FH and FBS had synergistic effects, and both were required for efficient cell proliferation, irrespective of the presence of PLL/heparin/bFGF/FN at the cell growth substrate.

Peripheral nerve sections were reacted with antibodies against neurofilament (NF) to label axons and anti-platelet-derived growth factor receptor (PDGFR) to identify fibroblasts. With this double staining, the nerve connective tissue ensheathments effectively showed intense PDGFR signal around the axonal fascicles (Figure 2a). Subsequently, PDGFR and S100 protein immunocytochemistry was used to obtain additional information on cell morphology and phenotypes growing from peripheral nerve cells cultured on PLL/heparin/bFGF/fibronectin-coated surfaces. In agreement with counts of Sox10^+^ cells (Section 2.1), at 3 DIV, most cells in vitro had bipolar shape and small size and were strongly positive for S100, which are typical characteristics of Schwann cells [44,50]. Consistent with the observation of large cell nuclei negative for Sox10 (Figure 1c), a scatter of cells frequently showed large amoeboid cytoplasm and intense staining for PDGFR (Figure 2b), likely representing a remnant of fibroblasts from the perineurium and endoneurium. Few fibroblast-like cells were observed in the cell cultures, irrespective of the cell culture media (Figure 2b).

### 2.3. Cell Growth from Peripheral Nerves on PEDOT-Coated Carbon Microfibers

Nerve explants were placed on the suspended microfibers to avoid contacting the bottom of the cell culture chamber. In this manner, we ruled out any influence of the glass surface on cell behavior and achieved a cell culture system that more closely reproduces the biomaterial–cell interactions expected in vivo after implantation of PCMFs in PNI models. On the other hand, 18 mm of microfiber length was available for cell growth from each nerve explant, in order to prevent complete coverage of the microfiber by migrating cells and, thus, avoid bias when analyzing the cellular effects of electrical stimulation and cell culture medium composition.

**Figure 3:**
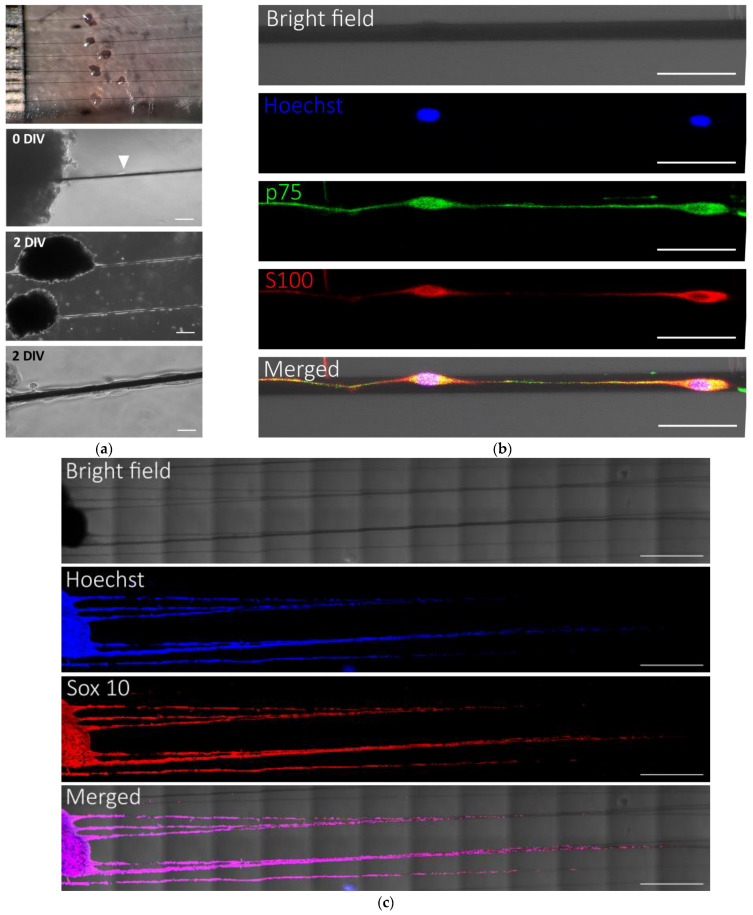
Cell growth from preconditioned sural nerves on PLL/heparin/bFGF/fibronectin-coated PCMFs. (**a**) Phase contrast microphotographs showing the appearance of nerve explants and migratory cells on PCMFs; Top panel: macroscopic appearance of a cell culture plate with PCMFs and recently attached nerve segments. Second panel: scarce cells (arrowhead) migrate on the PCMFs the first day in vitro (DIV). Third panel: migratory cell chain established by 2 DIV. Bottom panel: microphotograph at higher magnification illustrating the appearance of cells on the microfibers at 2 DIV. (**b**) Confocal immunofluorescence images of cells at the growth front showing positive staining for p75 (green) and S100 (red), which are the characteristic markers of Schwann cells. (**c**) Confocal microscopy images exemplifying peripheral nerve cell growth for 7 DIV on PCMFs. The cultures were fixed and processed for Sox10 immunocytochemistry combined with Hoechst nuclear staining. Scale bars: (**a**), 100 µm, 200 µm, 25 µm; (**b**), 50 µm (**c**) 1 mm.

Cells from fresh or preconditioned rat peripheral nerves, fed with NB/FBS/FH, avidly proliferated and migrated on PLL/heparin/bFGF/fibronectin-coated PCMFs. When fresh nerves successfully attached to the microfibers, there was no apparent difference regarding SC growth on the PCMFs compared to preconditioned nerves. However, whereas essentially all preconditioned nerves permanently adhered to the microfibers, more than half of the fresh nerves detached when adding cell culture medium or soon after. Thus, preconditioned nerves provided a much more reliable experimental system and were used in subsequent studies. Usually, the nerve cells lasted two days to extend processes and start migrating on PCMFs (Figure 3a) and, after that, moved at a rate of about 1 mm/day. The growing cell front on the microfibers was led by S100 and p75-positive SCs that extended long cytoplasmic processes (Figure 3b). This resulted in a chain of migratory cells of about 5 mm in length by 7 DIV (Figure 3c). The great majority (about 98%) of cells migrating on the microfibers were SCs, as attested by their immunoreactivity for Sox10 (Figure 3c).

**Figure 4:**
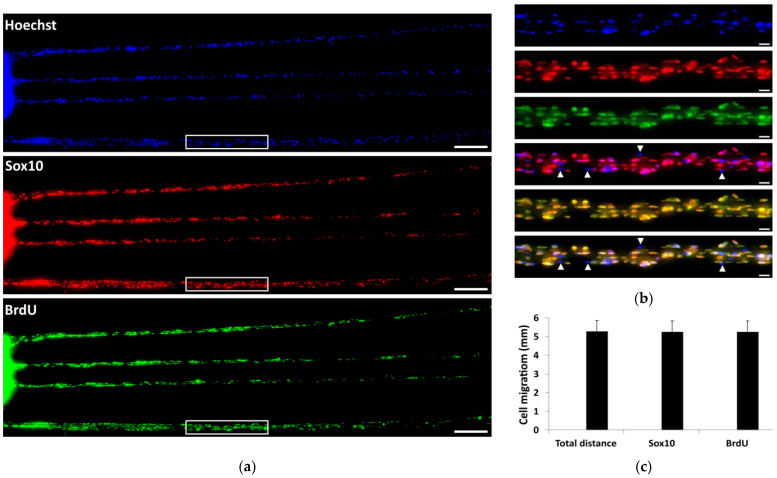
Cell proliferation from preconditioned rat nerves on PLL/heparin/bFGF/fibronectin-coated PCMFs. (**a**). Representative confocal fluorescence images of cells at 7 DIV, stained for Sox10, BrdU, and Hoechst. Proliferating (BrdU^+^) cells are distributed along the entire length of the microfibers. (**b**) Higher magnification of the boxed region in (**a**). Although most cells are proliferating SCs, a few Sox10-negative, non-dividing cells are also present on the PCMFs (arrowheads). (**c**) Average distance traveled by cells on PCMFs as assessed by the different markers. Scale bars: (**a**), 200 µm; (**b**) 20 µm.

Cellular uptake of BrdU was investigated to elucidate whether rat SCs proliferated on the PCMFs or only migrated from the peripheral nerve explants. After 7 DIV, BrdU^+^ cells were found all along the migratory chain in the microfibers up to the growing cell front, i.e., at the distal or farthest zone from the explant (Figure 4a,c), suggesting that cells actively divided on PCMFs. Most BrdU^+^ cells were positive for Sox10 (Figure 4a,b); i.e., they were SCs that entered the mitotic cycle. Sox10-negative cells usually showed no BrdU incorporation (Figure 4b), indicating that the cell culture conditions on the microfibers were optimal for SCs and did not favor the proliferation of fibroblasts or other cell types.

**Figure 5:**
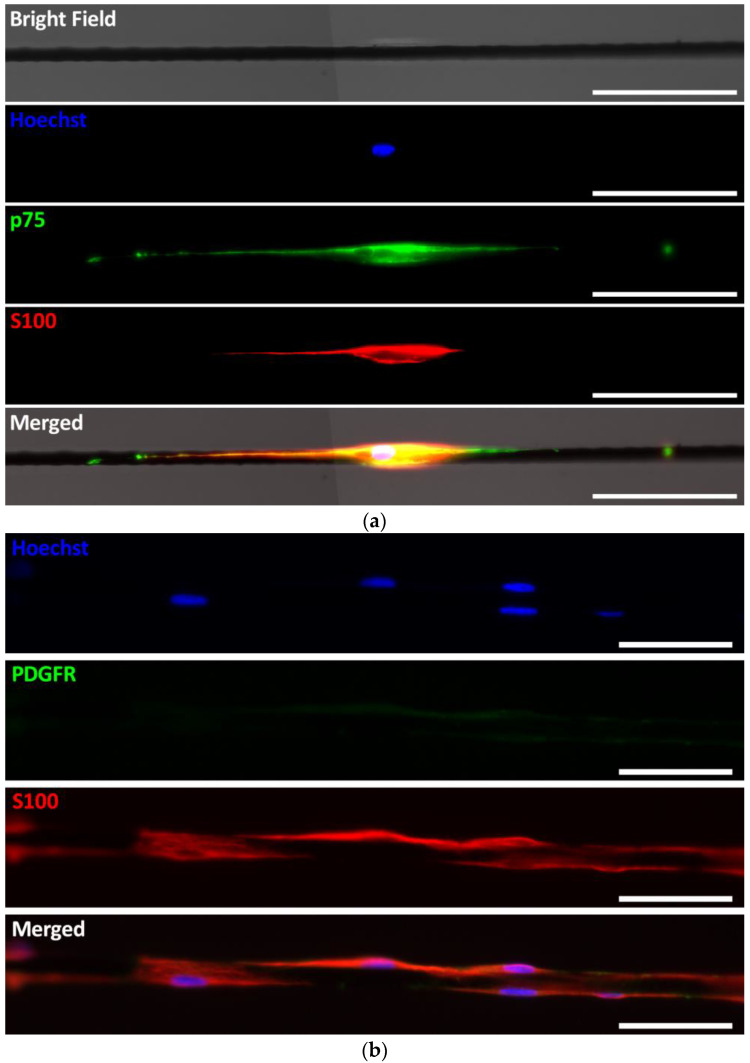
Immunostaining of Schwann cells from preconditioned porcine peripheral nerves growing on PLL/heparin/bFGF/fibronectin-coated PCMFs for 7 DIV. (**a**) Confocal fluorescence images of cells showing immunoreactivity for p75 (green) and S100 (red), the typical markers of SCs. (**b**) S100 (red) expressing SCs showing no labeling for fibroblast markers such as PDGFR (green). Scale bars: (**a**), 100 µm; (**b**) 50 µm.

The behavior of porcine cells was very similar to that of rat cells, avidly proliferating and migrating on PLL/heparin/bFGF/fibronectin-coated PCMFs. After some days necessary for appropriate nerve attachment to the microfibers, porcine SCs moved at about 1 mm/day and had traveled approximately 1 cm on the fibers by two weeks. As rat SCs, pig SCs expressed Sox10 and additional SC markers such as p75 and S100 (Figure 5). A minor percentage of cells on the microfibers was strongly stained for PDGFR and likely corresponded to fibroblasts, as mentioned above.

Despite the success in cultivating porcine nerves on suspended PCMFs, the large diameter of those nerves imposed some variability in the time needed to attach them stably on the fibers, and this variability translated to the measurements of cell migration taken for each explant during the first week of cell culture. Although porcine peripheral nerve cultures can be optimized by dissecting out specific nerve fascicles of smaller diameter, we opted for testing the effects of ES on the more reproducible, easy-to-handle, and readily available rat peripheral nerves.

### 2.4. Sub-Culturing Cells That Migrate Along PCMFs

**Figure 6:**
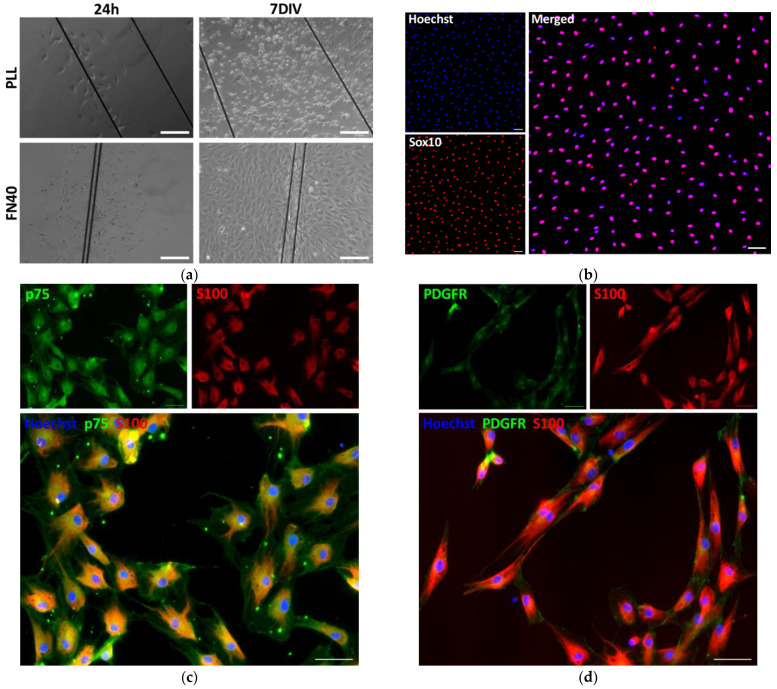
Sub-culture of cells migrating on PCMFs. Microfibers with attached cells were cut at 7 DIV and placed on plastic cell culture plates for an additional period of 7 days. (**a**) Representative phase-contrast microphotographs of cells after 1 or 7 days on plastic surfaces coated with PLL alone or with PLL/heparin/bFGF/fibronectin-40. (**b**) Cell immunostaining for Sox10 (red) at 7 DIV in PLL/heparin/bFGF/fibronectin-40 coated plates. (**c**,**d**) Confocal fluorescence images of cells processed for p75 (green) and S100 (red), or PDGFR (green) and S100 (red). Scale bars: (**a**), 200 µm; (**b**,**d**) 50 µm.

Cells migrating for seven days on PCMFs could be succesfully separated and sub-cultured. After cutting the parts of the fibers covered by cells and transferring them to standard polystyrene cell culture plates, the cells spontaneously migrated away and continued expanding on the flat plastic surfaces coated either with PLL alone or with PLL/heparin/bFGF/fibronectin (Figure 6a). Although only about 70% of SC purity was obtained after the additional period of 7 days on the plastic surfaces, the cells multiplied, had healthy appearance, and intensely stained for p75 and S100 (Figure 6b–d).

### 2.5. Cellular Effects of ES Through PCMFs and Synergies with Soluble Factors

Finally, biphasic ES was applied to rat peripheral nerves attached to PCMFs coated with PLL/heparin/bFGF/fibronectin. The possible synergisms among electrical stimuli, serum, and forskolin/heregulin on SC growth were also investigated.

**Figure 7:**
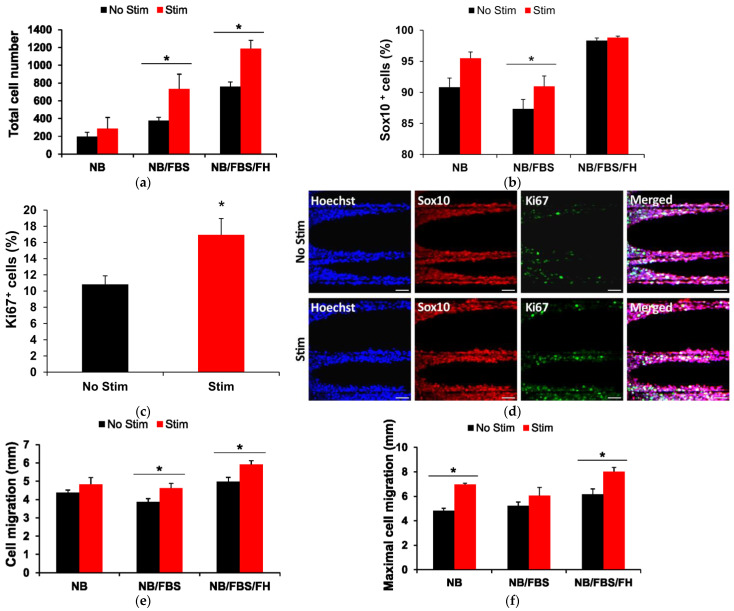
Biological effects of electrical stimulation (ES) applied through PCMFs to rat peripheral nerve cells. ES was initiated two days after placement of preconditioned sural nerves on PCMFs. Three different cell culture media were used for cell assessment, namely NB, NB/FBS, or NB/FBS/FH. Cells were fixed at 7 DIV and processed for immunocytochemistry. (**a**) Average number of migrating cells per microfiber in absence of stimulation (black columns) or after ES applied for 5 days (red columns). (**b**,**c**) Percentage of cells positive for Sox10 and Ki67, respectively. Quantification of Ki67-positive cells was performed only for cells in NB/FBS/FH. (**d**) Representative confocal fluorescence images of cell cultures analyzed in (**c**). (**e**,**f**) Mean (**e**) and maximal (**f**) cell migration per microfiber. Statistical significance was assessed using two-way ANOVA and Holm–Sidak post hoc test. Only the statistical differences detected when comparing stimulated and non-stimulated cells are indicated in the graphs. * *p* < 0.05. Scale bar in (**d**): 50 µm.

As expected, withdrawal of FBS and FH from the cell culture medium had a pronounced negative effect on cell proliferation on the PCMFs, strongly reducing total cell counts (two-way ANOVA, *p* < 0.001, Figure 7a). Adding FBS doubled cell numbers, and further supplementing with FH produced a four-fold cell multiplication compared to NB culture medium alone. ES further potentiated cell division on the microfibers, producing a marked, statitistically significant increase in cell counts in the presence of FBS or FBS/FH (Figure 7a). The enhancement in cell numbers by ES was mainly due to SC proliferation, as indicated by the higher percentage of Sox10-positive cells in stimulated cultures (Figure 7b) and of cells labelled for both Sox10 and Ki67 (Figure 7c,d).

Compared to cell proliferation, cell migration on PCMFs was much less dependent on the addition of FBS and FH to the culture medium (Figure 7e,f). Although showing low proliferation, cells on PCMFs still migrated, on average, more than 4 mm in NB alone, and no significant effect of ES was noted on mean migration values in this condition. FBS produced no enhancement in cell migration at all, whereas FBS/FH favored a subtle increase (~15%) that was doubled when ES was applied (Figure 7e). When maximal cell migration (i.e., the distance travelled by the cell at the tip of the migratory front) was compared, the effects of ES were somewhat more evident, with a 30–40% larger migration in the electrical-stimulated cells cutured with NB or NB/FBS/FH but not when fed with NB/FBS (Figure 7f).

## 3. Discussion

PCMFs support long-distance glial cell migration and axonal growth of CNS tissue in vitro [33,35] and facilitate CNS repair in vivo [38,39,40,41]. To initiate exploration of their usefulness for promoting PNS regeneration, the present work investigated their suitability as an electroactive substrate for SCs in vitro. After selecting the optimal molecular complex for microfiber surface modification and developing methods for peripheral nerve culture on self-standing microfibers, biofunctionalized PCMFs proved to be an excellent substrate for the attachment and directional extension of rodent and porcine SCs. Additionally, SC proliferation and migration could be substantially enhanced by electrical stimuli applied through the microfibers, and ES synergized with serum and molecules such as forskolin/heregulin in promoting SCs growth. These results provide a baseline for using biofunctionalized PCMFs for peripheral nerve repair and suggest the possibility of improving nerve regeneration through long tissue gaps by combining electroconducting microfibers and pharmaceuticals.

Nanofibers and microfibers, usually composed of extracellular matrix proteins or non-conducting polymers, are frequently used as substrates for peripheral neurons in culture [51,52,53]. Because nanofibers and many microfibers are not self-standing, they are deposited over sheets of other materials or at the bottom of cell culture plates that provide mechanical support to both the fibers and the cultured cells. In such an experimental paradigm, the fibers at the underlying support guide elongating axons, whereas SCs secondarily migrate, accompanying the axons [51,52,53], similarly to the behavior they have during PNS development [9,10]. Nevertheless, peripheral nerve regrowth follows a different dynamic in vivo, where SC proliferation and migration advantage axonal extension some days after injury, and the cells extend longitudinally to form bands of Büngner that help guide axonal elongation [8,16]. The major role that SCs play in peripheral nerve regeneration in vivo makes it of paramount importance to directly study their interactions with biomaterials aimed at serving as a scaffold for nerve repair, as well as their responses to electrical and molecular facilitators of nerve regrowth, as explored in the present work.

PCMFs have great potential for developing regenerative neuroprostheses [54]. They feature four-orders-of-magnitude higher electrical conductivity than conducting polymer composite conduits used in peripheral nerve repair [25], thus enabling reliable sensing and stimulation of neural tissue [55,56,57]. Additionally, their length is customizable to the neural gap, and they bear no risk of constriction axonopathy because the tissue grows centrifugally on the microfibers [54]. In addition to the PCMF platform, high-precision techniques are available for the fabrication of PEDOT micropatterned electrodes [58], support-free PEDOT microfibers [59], or PEDOT-hydrogel microfibers [60], which might also be suitable for use in implantable devices for bidirectional electrochemical communication with the nervous system [61]. Nevertheless, some hurdles must be solved to incorporate PEDOT-based scaffolds into devices for neural tissue regeneration. Despite their outstanding performance for recording neural electrical signals and stimulating neuronal activity in vitro [55] or acutely in vivo [56], using PCMFs for chronic ES in vivo has been hampered by their relatively low electrical and mechanical stability compared to platinum/iridium or other electrode materials commonly used in neuroprosthetic devices. PCMFs of short length (250 μm) were very effective in activating rat spinal motoneurons at low stimulus thresholds (≤50 μA) using biphasic electric current stimulation, but they deteriorated after a few thousand electric pulses [56]. Reducing the electric charge density applied per pulse (by increasing the microfiber length to 6–12 mm while administering the same current) extended the endurance of PCMFs and allowed for the application of about 1.5 million pulses in electrochemically safe conditions in vitro and after their implantation in the porcine spinal cord in vivo [57]. However, the PEDOT coating still showed signs of fracture and detachment from the carbon surface. Nevertheless, PCMFs-scaffolds are envisaged for the treatment of long neural injuries, thus requiring microfibers in the order of cm. This will further reduce the demand for electric charge density, consequently increasing the durability of the microfibers. On the other hand, electroactive covalent linkers have been developed to enhance PEDOT adhesion to the underlying carbon microfiber substrate, thus simultaneously improving the mechanical and electrical performance of PCMFs, as well as their integration in the neural milieu [62]. As a result, PCMFs are consolidating as a valuable component for combinatorial neurotherapeutics.

In alignment with previous studies on rodent and human SCs [45,48,49], we found that molecules coating the cell culture substrate and soluble factors added to the culture medium had a potent synergism on SC proliferation. Nevertheless, the differences between the behavior of SCs reported in the current study and that of CNS glial cells investigated in previous studies [35] are worthy of consideration. Glial precursor cells from the rodent cerebral cortex abundantly proliferated on PLL/Heparin/bFGF/FN-coated PCMFs, without the need to add serum or growth factors to NB cell culture medium [35]. In contrast, rodent SCs barely proliferated on the same multimolecular substrate after the withdrawal of FBS and FH from the culture medium, although they still migrated efficiently along the microfibers. This discrepancy is likely related to the age of the tissue source, because CNS glia was obtained from embryonic rats, whereas SCs originated from adult animals. Surface coating with PLL, laminin, or other biomolecules is sufficient for the adhesion of SCs in vitro [44]. SCs avidly migrate on fibronectin-coated substrata or fibronectin nanofibers [63,64], and the addition of fibronectin to engineered nerve conduits improves axonal regeneration in adult animals [17,65]. In addition to influencing cell migration, fibronectin also enhances SC proliferation [66]. bFGF similarly has mitogenic and pro-migratory activity in SCs [49,67] and positively influences peripheral nerve regeneration in vivo [68]. Therefore, it was not surprising that SCs migrated on PLL/heparin/bFGF/FN-coated PCMFs, even in the absence of FBS and FH, but their poor proliferation and even survival in this context were somewhat unexpected. However, the latter result is in resonance with the fact that bFGF acts as a survival factor only for Schwann cell precursors but not for postnatal SCs and that the action of bFGF as a survival factor for postnatal SCs depends on the presence of forskolin or other cAMP-stimulating molecules [49]. In light of these considerations, embryonic SCs are predicted to be less demanding for serum and FH for their survival and proliferation on PLL/heparin/bFGF/FN-coated microfibers.

For the same cell culture conditions, rat SCs multiply at a substantially higher rate than human cells [69]. Peripheral nerve regeneration also progresses at a slower rate in humans [7], although the reasons for this are not completely understood. Similar to the case of SCI [41,70], large animal models of PNI [71,72,73,74,75] have been proposed to test lesion bridging devices in a biological context more similar to that of humans. In this regard, the use of pigs is continuously extending with significant advances reported for long-distance regeneration of porcine nerves [71,73] and efficient culture and expansion of porcine SCs [44,76]. With the aim of enhancing the translational relevance of our results, we tested the suitability of PLL/heparin/bFGF/FN-coated cell culture plates and PCMFs to support the growth of porcine SCs. Similar to the case of rat SCs, the mentioned multimolecular complex enabled excellent proliferation and migration of porcine SCs and was synergistic with FBS/FH. Rat and human SCs differ not only in proliferation rate but also in their responsiveness to growth factors, the expression of cell membrane receptors for growth factors and extracellular matrix proteins, and their ability to interact with axons [69]. Scarce information is available regarding the specific biology of porcine SCs, but it is known that they share some phenotypic characteristics with rat and human SCs. For instance, similar to rat SCs, porcine cells robustly myelinate axons from their own species, and similar to human SCs, they are more dependent on neuregulin as a trophic factor [76]. Although we performed no comparison of the differential molecular profiles of rat and pig SCs, our results showed that in the absence of serum and FH, porcine cells survived to a lower extent than rodent cells on the PLL/heparin/bFGF/FN-coated substrate. Further studies are needed to investigate the reasons for the higher dependency of porcine SCs for trophic support and the relevance of this finding for human neuropathology. On the other hand, our assessment of ES was limited to rat SCs, and, therefore, the findings regarding the electrical enhancement in cell proliferation need confirmation in porcine and human cells. Nevertheless, the results suggest that PLL/heparin/bFGF/FN is appropriate for microfiber modification and implantation at injured human nerves.

Biphasic ES can either promote [24] or inhibit [77] the proliferation of SCs in vitro as a function of the applied electrical parameters. In vitro, pro-mitotic ES protocols simultaneously increase SCs’ production of neurotrophic factors such as NGF, BDNF and GDNF [22,23,24], and electrically stimulated SCs promote the extension of neuronal processes of unstimulated neurons [78]. Most frequently, voltage ES is applied by electrodes immersed in the cell culture medium rather than through the cell-growing substrate. In voltage stimulation, the intensity and time decay of the electric current are usually unknown and will vary with the technical specifications of the electrodes and cell culture chamber. Current-controlled ES ensures current flow for the programmed time and intensity, which is more appropriate for physiological investigations but has been infrequently used to enhance SC biology [24]. Nevertheless, our results are in line with the latter study [24], in which SCs doubled cell proliferation when charge-balanced, and biphasic ES was applied for seven days through gold substrates. To advance in the goal of developing an implantable electroactive scaffold for peripheral nerve repair, we applied charge-balanced, biphasic ES 6 h day for five days through self-standing PCMFs that served both as an electrode and SC substrate. In this paradigm, ES increased cell proliferation and migration and synergized with biomolecules at the microfiber surface and cell culture medium.

Although ES is known to have direct effects on sensory and motor neurons that enhance axon regeneration in vivo [7], for instance, upregulating GAP-43 and BDNF and its receptor trkB [20,21], the possibility exists that other cell types such as SCs and even fibroblasts from the nerve ensheathments are activated by ES and contribute to the nerve growth response. The demonstration of enhanced cell proliferation and growth factor release by ES of SCs [22,23,24], together with our current findings regarding electrically increased SC proliferation and migration on PCMFs, suggests that the implantation of electroactive microfibers will substantially enhance peripheral axonal elongation in vivo. Furthermore, the fact that ES through PCMFs positively impacted SC biology without favoring fibroblast expansion may be advantageously used for long-gap nerve repair, where fibrosis markedly reduces the chances of successful axonal regrowth [79]. Most importantly, by decorating subsets of microfibers with specific cell adhesion molecules and applying sub-chronic or long-term ES, the microfiber-based nerve scaffold may allow for a more selective and sustained control of axonal regeneration through and beyond the nerve gap while simultaneously providing a useful tool for sensorimotor rehabilitation. This dual function of PMCFs as electroactive pro-regenerative scaffold and neuroprosthetic electrode for driving neural activity might facilitate muscle reinnervation and prevent muscle atrophy, thus improving the long-term neurological outcome of people afflicted with PNI.

## 4. Materials and Methods

### 4.1. Selection of Molecules for Substrate Coating

Poly-L-Lysine (PLL) and laminin are frequently used to coat cell culture plates for expanding SCs in vitro [44], whereas fibronectin and bFGF are mitogenic and have pro-migratory activity for SCs [63,64,65,66,67], and a complex of PLL/heparin/bFGF/fibronectin provides an optimal substrate for glial cell precursors from the central nervous system [35]. Therefore, we compared those substrate coatings with the aim of selecting the optimal molecules for promoting SC proliferation and migration on PCMFs. To expedite the selection, dissociated cells from peripheral nerves were seeded in polystyrene cell culture plates pre-treated with either (1) PLL alone; (2) PLL/Laminin (Thermo Fischer Scientific, Waltham, MA, USA; A29248); (3) PLL/heparin/basic fibroblast growth factor (bFGF)/Fibronectin 20 µg/mL, referred in the text as FN20; or (4) PLL/heparin/bFGF/fibronectin 40 µg/mL, referred to as FN40.

PLL (Sigma-Aldrich, St. Louis, MO, USA; P2636) was applied at 45 µg/mL in water, for 1 h at RT. The same procedure was used for the four surface treatments. For protocol (2), the PLL-coated plates were further incubated with 1 µg/cm^2^ laminin overnight at 37 °C. For coatings (3) and (4), the PLL-coated plates were covered with a solution of 5.4 mg/mL heparin (Sigma-Aldrich, St. Louis, MO, USA; H5515) in phosphate buffered saline (PBS; Sigma-Aldrich, St. Louis, MO, USA; H5515) for 4 min at RT. Subsequently, recombinant human bFGF (R&D Systems, Minneapolis, MN, USA; 4114-TC-01-M) was applied at 1 mg/mL in 0.1 M PBS for 1 h. Finally, the culture plates were incubated for 4 days at 37 °C in PBS containing 20 or 40 µg/mL bovine fibronectin (Sigma-Aldrich, St. Louis, MO, USA; F1141). The plates were always washed with PBS, three times for 5 min each, after the application of each molecule.

### 4.2. Preparation of Cell Culture Chambers Containing Biofunctionalized PCMFs

Cell culture chambers containing aligned, suspended (i.e., elevated from the bottom) PCMFs of about 18 mm in length were fabricated following published protocols [33,35]. In brief, the carbon microfibers (7 µm diameter, Goodfellow, Huntingdon, UK) were stuck on two Teflon sheets (0.7 mm high) placed at the borders of a borosilicate glass. A 4-well polystyrene chamber (Corning, NY, USA; 354114) of 11 × 11 × 20 mm width, height and depth, respectively, was bonded to the glass and the Teflon with medical-grade silicone (MED-4210; Nusil, Carpinteria, CA, USA). The carbon microfibers exited one side of the cell culture chamber and ended in a conductive carbon tape that was interconnected to an Autolab PGSTAT302 potentiostat/galvanostat (Eco Chemie, Utrecht, The Netherlands). PEDOT:PSS-*co*-MA was electropolymerized on the microfibers by applying a constant current of 1 µA/mm^2^ and a charge density of 96 mC/cm^2^. The resulting PCMFs were sterilized with paraformaldehyde gas and subsequently modified with the multimolecular complex of PLL/heparin/bFGF/fibronectin-40, which produced the best results for SC expansion on standard cell culture plates (Section 2.1). For PCMFs, PLL was covalently bonded to the carboxylic groups of PSS-*co*-MA using EDC/NHS (Sigma-Aldrich, St. Louis, MO, USA) [33,35]. The other molecules were applied as for standard cell culture plates (Section 4.1).

### 4.3. Peripheral Nerve Sources

**Figure 8:**
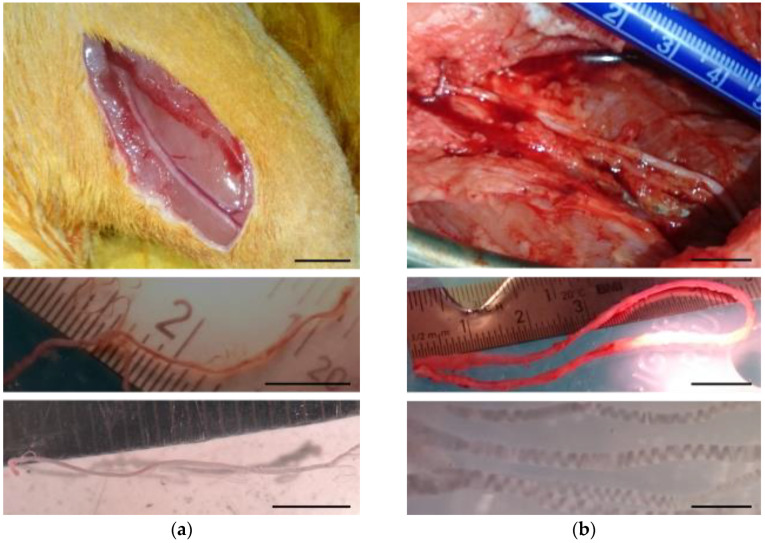
Photographs illustrating the dissection of sural nerves from rats (**a**) and pigs (**b**). The upper panels show sural nerve exposition by a dorsal longitudinal incision from the popliteal fossa to the lateral malleolus. The extracted nerves, still with the perineurium and epineurium, are shown in the middle panels, while the sural nerve fascicles devoid from the epineurium and perineurium appear at bottom.

Adult male Wistar rats (14–20 weeks old, 300–390 g) raised at the animal facility of the Hospital Nacional de Parapléjicos were used. The rats were housed in groups of two for a 12 h light/dark cycle with food and water available ad libitum. General anesthesia was obtained with intraperitoneally applied sodium pentobarbital (Vetoquinol, S.A, Lure, France; 55 mg/kg) mixed with atropine (B. Braun, Jaen, Spain; 0.025 mg/kg) and xylazine (Calier, Barcelona, Spain; 5 mg/kg). Both hind legs were incised, and the sural nerves were dissected using a stereoscopic microscope (Leica Biosystems Nussloch GmbH, Nussloch, Germany) (Figure 8a). About 4 cm of nerve was extracted and placed in cold Hank’s balanced salts solution (HBSS, (Sigma-Aldrich, St. Louis, MO, USA; H6648). The sural nerve was selected as cell source because it is the nerve from which human Schwann cells are most commonly harvested for clinical trials in neural repair [80], and this will allow for future comparisons of the current data with those obtained on human SCs.

Sural nerves were also obtained from 2-month-old Large White female pigs (*Sus scrofa domesticus*) purchased from a commercial supplier (Granja Agropardal, Toledo, Spain). In this case, the surgical procedures were performed under inhalational anesthesia. Anesthesia was induced by intramuscular (IM) injection of ketamine (Eurovet Animal Health B.V., Bladel, The Netherlands; 10 mg/kg), midazolam (Laboratorios Normon, S.A., Madrid, Spain; 0.1 mg/kg), and medetomidine (Laboratorios Syva, S.A., León, Spain; 0.02 mg/kg), followed by intravenous (IV) administration of propofol (UAB Norameda, Klaipeda, Lithuania; 3 mg/kg). Then, a tracheal tube was placed, and the anesthesia was maintained with sevoflurane (AbbVie S.r.l, Campoverde di Aprilia, Italia; 1.7–2%) together with remifentanil (Laboratorio Reig Jofré, S.A., Barcelona, Spain; 26 mg/kg/h IV) and rocuronium (N.V. Organon, Oss, Netherlands; 1.2 mg/kg/h IV). Mechanical ventilation (Fabius Tiro, Dräger, Lübeck, Germany) was set at 12–14 breaths/min with a tidal volume of 10–15 mL/kg. Heart rate, blood pressure, exhaled carbon dioxide, blood oxygen saturation, and inspired and expired sevoflurane levels were monitored (Infinity Delta, Dräger, Lübeck, Germany). About 10 cm of nerve was removed and placed in cold HBSS (Figure 8b).

### 4.4. Nerve Processing and Cell Culture Media

#### 4.4.1. Dissociated SC Culture

Sural nerve fascicles were pulled from the epineurium and perineurium sheaths and cut into segments of approximately 2 mm in length. Then, they were transferred to 6-well cell culture plates and incubated at 37 °C with 5% CO_2_ for six days in Neurobasal (Thermo Fischer Scientific, Waltham, MA, USA; 21103049) supplemented with 0.5 mM L-glutamine (Sigma, St. Louis, MO, USA; G8540), 50U/mL Penicillin-Streptomycin (Thermo Fischer Scientific, Waltham, MA, USA; 15070063), 50 µg/mL Gentamicin, 10% fetal bovine serum (FBS, Thermo Fischer Scientific, Waltham, MA, USA; 10108-165), 1x B-27 (Thermo Fischer Scientific, Waltham, MA, USA; 0080085SA), 1 µM forskolin (Sigma-Aldrich, St. Louis, MO, USA; F3917), and 50 ng/mL recombinant human heregulin β-1 (Peprotech, London, UK; 100-03). The cell culture medium was changed every two days.

After six days in vitro (DIV), the nerve fascicles were transferred to a new well containing a cell dissociation medium comprising DMEM with high glucose (Biowest, Nuaillé, France, L0104-500), 3.1 mM CaCl_2_ (Sigma-Aldrich, St. Louis, MO, USA; 102382), 2U/mL Dispase II (Roche, Basel, Switzerland, 4942078001), and 0.1 mg/mL collagenase (Sigma-Aldrich, St. Louis, MO, USA; C9722) and placed in the incubator. The following day, the fascicles were centrifuged at 200× *g* for 5 min, and the cells were dissociated by pipetting and plated at a density of 5000 cells/cm^2^ on polystyrene cell culture plates (Thermo Fischer Scientific, Waltham, MA, USA, FB63186) pre-coated with cell attachment molecules, as described in Section 4.1. The cells were allowed to grow for 3 DIV in the same medium used for the preconditioning period and subsequently fixed with paraformaldehyde (Panreac, Barcelona, Spain; 211511) and processed for immunostaining. 5-Bromo 2-deoxyUridine (BrdU) (5 µg/mL, Sigma-Aldrich, St. Louis, MO, USA; B5002) was added to some cultures at 2 DIV to assess cell proliferation.

In complementary experiments, the cells were seeded on plates coated with PLL/heparin/bFGF/fibronectin-40 and fed only with NB medium, i.e., Neurobasal with L-glutamine, B-27, and antibiotics, as described above, or with NB supplemented with forskolin and heregulin (NB/FH) but not FBS. The latter experiments aimed at investigating if adding the multimolecular complex (PLL/heparin/bFGF/fibronectin) at the cell substrate was able to compensate for the absence of FH or 10% FBS on cell survival and growth.

#### 4.4.2. Nerves Cultured on PCMFs

Fresh or preconditioned sural nerve segments were individually placed on PCMFs functionalized with PLL/heparin/bFGF/fibronectin-40. Due to myelin and axon degeneration in the preconditioning period, each segment measured 1 mm or less when transferred to the well with suspended microfibers. Care was taken to place the nerve only on the fibers to avoid any contact with the glass at the bottom of the well. Thus, 5–7 nerve segments were cultured per well (Figure 2a). For the initial experiments, complete cell culture medium (NB/FBS/FH) was used. For experiments investigating the effects of ES, additional cell cultures were fed with media missing FBS and/or FH in order to investigate the possible synergisms between ES and soluble factors (Section 4.5).

#### 4.4.3. Secondary Culture of the Cells Migrating on PCMFs

To investigate if PCMFs can be useful to isolate cells migrating from the peripheral nerves, the parts of the microfibers covered by migratory cells were cut and separated from the nerve explants. The cut PCMFs with cells were then placed in polystyrene cell culture plates coated with PLL/heparin/bFGF/fibronectin-40 and were incubated with complete cell culture medium for 7 days to allow spontaneous cell detachment from the microfibers and proliferation on the polystyrene surface.

### 4.5. Electrical Stimulation

Cell culture chambers were prepared as described in Section 4.2 and further elaborated for ES through the PCMFs. For this, the microfibers exiting one side of the cell culture wells were interconnected to a stainless-steel microwire (A-M Systems 793400, Sequim, WA, USA) using carbon tape and colloidal graphite (Agar Scientific, Stansted, UK). Silicone was cured on top of the interconnection for electrical insulation. A second stainless-steel microwire, with 8 mm of the insulating layer peeled off, was inserted on the opposite wall of the cell culture plate orthogonal and 7 mm elevated from the PCMFs. The microfibers and the counter-electrode were immersed within 1.5 mL of cell culture medium, and the stainless-steel microwires were interfaced to an electric pulse generator (Biologic VSP20, Seyssinet-Pariset, France) outside the cell incubator.

Preconditioned rat sural nerves were carefully placed on the PCMFs functionalized with PLL/heparin/bFGF/fibronectin-40. The explants were located only on one side of the cell culture wells, close to the entry point of the electric current. ES started two days after introducing the nerves to allow for cell adhesion to the microfibers. During those two days, the nerves were kept in complete cell culture medium, i.e., NB/FBS/FH. This medium was replaced by either NB, NB/FBS, or fresh NB/FBS/FH just before starting the stimulation at 2 DIV, in order to discern the synergistic effects that the ES protocol might have with serum and the soluble factors (FH). The cell culture medium was not further replaced during the five days of stimulation. A sequence of 15 charge-balanced biphasic electric pulses, with a cathodic phase of −100 µA/200 µs, followed by an anodic phase of +50 µA/400 µs, was applied through the PCMFs during 45 ms. This train of stimuli was repeated each 2 s, 6 h/day for 5 days. Electrically stimulated peripheral nerve explants and their respective non-stimulated controls were fixed at 7 DIV, just after ending the five-day stimulation protocol.

### 4.6. Immunocytochemistry and Cell Quantification

Paraformaldehyde (2%) was added to the cell culture medium for cell fixation, and immunofluorescent labeling combined with Hoechst 33342 (Molecular Probes, Eugene, OR, USA) nuclear staining was used to identify SCs and study cell migration and proliferation. Antigen retrieval for Sox10, BrdU, and Ki67 was achieved by immersing the fixed cells for 45 min in MQ water with sodium citrate (Sigma-Aldrich, St. Louis, MO, USA; S1804) 0.01 M, pH 6, at 90 °C. Subsequently, the cells were incubated for 1 h in 0.1 M, pH 7.4 PBS containing 0.2% Triton (Panreac, Barcelona, Spain; 142314) and 5% normal goat serum (NGS, Sigma-Aldrich, St Louis, MO, USA; NS02L), rinsed with PBS and then immersed overnight at 4 °C in combinations of antibodies raised against: Sox10 (Abcam AB212843, Cambridge, UK; 1:1000), S100 (Sigma-Aldrich, St Louis, MO, USA; S-2532; 1:1000), p75 (Millipore AB1554, Burlington, MA, USA, 1:500), PDGFR (Abcam AB32570; 1:100), BrdU (MoBU-1, Alexa Fluor™ 488-labeled, Invitrogen B35130, Waltham, MA, USA, 1:100), or Ki67 (Thermo Fischer Scientific RM-9106-S0, Waltham, MA, USA, 1:200). Furthermore, Hoechst 33342 (Molecular Probes) was applied at 1 µg/mL in PBS for 15 min. On the next day, the cultures were incubated for 1 h in a PBS solution containing anti-rabbit IgG or anti-mouse IgG antibodies labeled with Alexa-488 or Alexa-594 (Molecular Probes, Eugene, OR, USA; 1:700), respectively. Controls without the primary or secondary antibodies were included in all the immunostaining procedures.

For cell quantification on plastic surfaces, 25 images of each well of 96-well culture plates were automatically captured with a 20× objective using a high-content screening (HCS) system consisting of an Olympus 1X83 microscope (Olympus, Tokyo, Japan) equipped with a digital camera (Orca-Flash 4.0, Hamamatsu Photonics, Hamamatsu, Japan), controlled by the dedicated HCS acquisition software scanR 3.5.0 (Olympus, Tokyo, Japan). Prior to cell nuclei identification and quantification, a deep learning-based neural network model, integrated into the scanR analysis software, was trained to accurately recognize and identify each cell nucleus. Nuclei were subsequently segmented and counted using this model. Sox10- and BrdU-positive cells were identified by assessing the co-localization of marker-specific fluorescence signals with previously detected nuclei.

Cells on the microfibers were imaged with an SP5 confocal laser-scanning microscope (Leica Biosystems Nussloch GmbH, Germany) equipped with resonant scanner, Argon and HeNe lasers for excitation of Alexa-labeled antibodies, and a 405 nm diode laser to excite Hoechst. The MFs were simultaneously imaged by transmitted light. To visualize cells along several mm of microfiber, Z-stacks of images taken each 7 µm were obtained with a 10× objective. The LAS AF Lite software (v. 1.9, Leica Microsystems CMS GmbH, Germany) enabled automatic acquisition of image series spanning the length of microfiber covered by the nerve explant and migratory cells and the construction of image mosaics that were used to quantify the following parameters: (1) distance traveled by SCs (cell nuclei labeled with Hoechst and positive for Sox10) on the MFs; (2) number of SCs on the MFs; and (3) number of dividing cells (Ki67+ or BrdU + SCs). In the same confocal microscope, zoom of 4 with 6-micron step-size was used when needed for improving cell visualization at the proximal, medium, or distal part of the microfiber.

### 4.7. Statistics

Statistical analyses were performed with the SigmaStat 4.0 software (Systat Software, Chicago, IL, USA). All values reported, unless otherwise stated, are means ± standard error of the mean (SEM). All data groups were examined for normality using the Kolmogorov–Smirnov test. Two-way ANOVA, followed by the Holm–Sidak posttest, was used to compare the average values of the effects of the cell substrate, electrical stimulation and soluble factors on cell biology. Comparisons involving only two groups, for instance, the quantification of Ki67-positive cells on stimulated and non-stimulated cell cultures, were performed with a *t*-test. Differences were considered statistically significant at *p* < 0.05.

## 5. Conclusions

This study demonstrates that biofunctionalized, PEDOT-coated carbon microfibers can be advantageously used as substrates for promoting long-distance, directed migration of Schwann cells from rodent and porcine peripheral nerves. The application of charge-balanced biphasic electric current pulses through the microfibers enhanced SC proliferation and migration in vitro, without favoring fibroblast overexpansion. ES was synergistic with the action of biomolecules (PLL/Heparin/bFGF/FN) at the cell substrate, and soluble factors added to the cell culture medium (serum, forskolin, heregulin). These data provide a baseline for using PCMFs as an electroactive scaffold for bridging peripheral nerve gaps and suggest that a therapeutic approach combining microfibers, ES, and pharmaceuticals will provide untapped opportunities for inducing functional peripheral axon regeneration and muscle reinnervation. In vivo studies in large animal models are warranted to assess the effectiveness of combinatory PCMFs/ES/pharmaceutical therapies. Additionally, future research will test the hypothesis that a similar electroceutical approach may persuade human SCs to myelinate regenerated axons, thus overcoming a major limitation of human cells and accelerating the clinical translation of this technology.

## Data Availability

The data presented in this study are available on request from the corresponding author.

## Abbreviations

The following abbreviations are used in this manuscript:

bFGF	Basic fibroblast growth factor
BrdU	5-Bromo-2′-desoxiuridine
DIV	Days in vitro
EDC	N-(3-Dimethylaminopropyl)-N′-ethylcarbodiimide
ES	Electrical stimulation
FBS	Fetal bovine serum
FH	Forskolin and heregulin β-1
HBSS	Hank’s balanced salts solution
NB	Neurobasal cell culture medium with L-glutamine, Penicillin-Streptomycin, Gentamicin, and B27
NHS	N-Hydroxysuccinimide
PBS	Phosphate buffered saline
PCMFs	Carbon microfibers coated with PEDOT doped with PSS-*co*-MA
PDGFR	Platelet derived growth factor receptor
PEDOT	Poly(3,4-ethylenedioxythiophene)
PLL	Poly-L-lysine
PNI	Peripheral nerve injury
PSS-co-MA	Poly [(4-styrenesulfonic acid)-*co*-(maleic acid)]
RT	Room temperature
SCs	Schwann cells
